# Fertility Inhibitors Macrocyclic Complexes of Bivalent Manganese: Synthetic, Spectroscopic and Medicinal Approach

**DOI:** 10.1155/S1565363303000190

**Published:** 2003

**Authors:** R. V. Singh, Ashu Chaudhary, Anita Phor

**Affiliations:** 1 Department of Chemistry University of Rajasthan Jaipur 302004 India; 2 Hindu College Sonipat 131001 India

## Abstract

The modern physico-chemical, spectroscopic and biochemical methods have proved an important tool to elucidate the constitution of transition metal complexes. This paper presents a brief account of the synthesis, spectroscopic and medicinal aspects of tetraazamacrocyclic compounds of manganese(II). Sixteen to eighteen membered tetraamide macrocyclic ligands DTTD^1^ and DTTD^2^ have been synthesized by the condensation of 1,2-diaminoethane and 1,3-diaminopropane with phthalic acid in the presence of condensing reagents dicyclohexylcarbodiimide and 4-dimethylaminopyridine. On reduction these macrocyclic ligands give new tetraazamacrocycles TTD^1^ and TTD^2^ which form complexes with manganese (II) nitrate and manganese (II) acetate. Based on chemical analyses, molecular weight determinations, conductance measurements, magnetic moment, IR spectra, ^1^H NMR, ^13^C NMR spectra, electronic spectra, mass spectra and X-ray spectral analysis, an octahedral geometry has been assigned to the newly synthesized products. The formulation of the complexes of the type [Mn(TTD^n^)X_2_] [where, n or 2, X (NO_3_) or (CH_3_COO)] has been established on the basis of chemical composition. The possibilities of potential uses of these complexes as fungicides and bactericides, studied in vitro, are also discussed. The testicular sperm density, sperm morphology, sperm mortality, density of cauda epididymis, spermatozoa and fertility in mating trials and biochemical parameters of reproductive organs of rat have been examined and discussed.


					Fertility Inhibitors Macrocyclic Complexes of Bivalent
Manganese : Synthetic, Spectroscopic and Medicinal
Approach
R.V. Singh*, Ashu Chaudhary and Anita Phor
1Department ofChemistry University ofRajasthan, Jaipur-302004, India
-Hindu College Sonipat 131001, India
ABSTRACT
The modern physico-chemical, spectroscopic and biochemical methods have proved an important tool to
elucidate the constitution of transition metal complexes. This paper presents a brief account of the synthesis,
spectroscopic and medicinal aspects oftetraazamacrocyclic compounds ofmanganese(II). Sixteen to eighteen
membered tetraamide macrocyclic ligands DTTD and DTTD have been synthesized by the condensation of
1,2-diaminoethane and 1,3-diaminopropane with phthalic acid in the presence of condensing reagents
dicyclohexylcarbodiimide and 4-dimethylaminopyridine. On reduction these macrocyclic ligands give new
tetraazamacrocycles TTD and TTD which form complexes with manganese (II) nitrate and manganese (II)
acetate. Based on chemical analyses, molecular weight determinations, conductance measurements, magnetic
moment, IR spectra, 1H NMR, 3C NMR spectra, electronic spectra, mass spectra and X-ray spectral analysis,
an octahedral geometry has been assigned to the newly synthesized products. The formulation of the
complexes of the type [Mn(TTDn)X2] [where, n or 2, X (NO3) or (CHCOO)] has been established on
the basis of chemical composition. The possibilities of potential uses of these complexes as fungicides and
bactericides, studied in vitro, are also discussed. The testicular sperm density, sperm morphology, sperm
mortality, density of cauda epididymis, spermatozoa and fertility in mating trials and biochemical parameters
ofreproductive organs ofrat have been examined and discussed.
INTRODUCTION
The chemistry of macrocyclic complexes has received much attention and such compounds have been
extensively studied in recent years /1/. Dramatic progress has been achieved in the field of macrocyclic
chemistry on account of its various applications/2/in bioinorganic chemistry. Catalysis, extraction of metal
ions from solution and the activation of small molecules gave impetus to this endeavor. In view of the
*Author for Correspondence E-mail kudiwal@datainfosys.net; Fax +91 (141) 2708621
233
Vol. l, Nos. 3-4, 2003 Fertility Inhibitors Macrocyclic Complexes ofBivalent Manganese
Synthetic, S.pectroscopic and.Medicinal Approach
presence of two possible potential donor atoms, viz nitrogen and oxygen, the coordination chemistry of
amide macrocyclic deserves special attention. It has been shown that amide macrocyclic compounds bear the
dual structural features of macrocyclic tetraamines and oligopeptides and can stabilize higher oxidation states
in some of the metal ions/3/. There are many examples of macrocyclic synthesis, mixtures of two or more
donor sites have also been employed to tune of the selectivity and stability/4/. The current interest is often
inspired by some other applications/5/and importance in the development of industrial area/6/.
Transition metals have aroused great interest in researchers, due to their biopotency, unusual stereo-and
magneto-chemistry and ability to form multiple complexes. The eagerness to reveal the characteristics of
transition metals has led to innovations in the area of model compounds/7-9/, in enzymatic mechanism/10-11/,
noble stoichiometric /12/ and catalytic oxygen transfer systems. The complexes of manganese play an
important role in photochemical reactions /13,14/. Several manganese complexes are known to exhibit
antifungal /15/ and antileukemic activities. In view of these interesting aspects we have synthesized and
characterized the tetraazamacrocyclic complexes of manganese(If) in the presence of
dicyclohexylcarbodiimide and 4-dimethylaminopyridine as condensing reagents for the condensation of 1,2-
diaminoethane, 1,3-diaminopropane and phthalic acid. The present paper deals with the striking structural
features, synthesis and appreciable biological applications ofthese complexes.
EXPERIMENTAL
The chemicals including dicyclohexylcarbodiimide and 4-dimethylaminopyridine and phthalic acid (Fluka)
were used as such. 1,2-Diaminoethane, 1,3-diaminopropane and LiAIH4 were used as obtained from
E.Merck. Mn(NO3)z.4H20 and Mn(CH3COO)2.4H20 were used without further purification.
Synthesis of the Ligands:
3,4 11, 12-Dibenzo-2,5,1O,13-tetraoxo-l,6,9,14-tetraazacyclohexadecane (DTTDI)
The reaction is carried out in 2 2 molar ratios of 1,2-diaminoethane and phthalic acid. A catalytic amount of 4-
dimethylaminophyridine and an appropriate amount of dicyclohexylcarbodiimide (1.3274g;6.43mmol) in a
minimum amount of dichloromethane at 0C were kept magnetically stirred in a two-necked round bottom
100 mL flask. The reaction was followed by the addition of 1,2-diaminoethane (0.3866 g; 6.43 mmol) and
phthalic acid (1.0681g; 6.43 retool) in dichloromethane. The resulting reaction mixture was stirred for about
10-12 hours at 0C. The solid product was obtained by filtration and washing with dichloromethane. The
white product thus obtained was recrystallised from benzene and dried in vacuo.
3,4:12,13-Dibenzo-2,5,11,14-tetraoxo-1,6,1O,15-tetrazacyclooctadecane (DTTD)
Similarly DTTD was prepared by reacting (1.0750g; 5.21 mmol), dicyclohexylcarbodiimide, (0.3868g;
5.21retool), 1,3-diaminopropane and (0.8662 g; 5.21 mmol) phthalic acid.
234
R. V. Singh et al. Bioinorganic Chem&try andApplications
1,6,9,14-Tetrazacyclohexadecane (TTD)
The reaction is carried out in 2 molar ratios of ligand DTTD and LiAIH4. The ligand DTTD (0.8765
g; 2.30 mmol) was dissolved in tetrahydrofuran and cooled at 0C. LiA1H4 (corresponding to DTTD) in
tetrahydrofuran was stirred for about 10 hours in an ice bath. The reaction mixture was then stirred under
reflex for 75 hours. After cooling it, l0 mL of water and 10 mL of 15% aqueous sodium hydroxide were
added to the reaction mixture at 0C. The solid material was filtered and the residue washed with
tetrahydrofuran Fhe filtrate and tetrahydrofuran washing were concentrated under reduced pressure to give
TTD as liquid.
1,6,10,15-Tetraazacyclooctadecane (TTD2)
The ligand TTD was synthesized analogous to the above ligand. The ligand DTTD (0.8216 g; 2.01
mmol) and LiA1H4 (0.1528 g; 4.02 mmol) were used for this synthesis.
Synthesis of the Complexes
Dinitrato/acetato(1,6,9,14-tetraazcyclolteradecane)manganese(ll)ln(TTDl)X/
The reaction is carried out in a molar ratio of ligand TTD and metal salt. To a methanolic solution
of metal salt (0.9467 g; 3.77 mmol) was added a methanolic solution of TTD (corresponding to metal salt).
The reaction mixture was stirred for about 15-18 hours. The resultant solid product obtained was filtel'ed off
and washed with methanol and dried in vacuo.
Dhtitrato/acetato (1,6,1015-tetraazacyclooctadecane) manganese (II)/Mn(TTD2)Xy':
These complexes were prepared using the above mentioned method for/Mn(TTD1)X2/. Ligand TTD was
used in this synthesis in place ofthe ligand TTD.
Physical Measurements and Analytical Methods
The molecular weights were determined by the Rast Camphor Method. Conductivity measurements in dry
dimethylformamide were performed with a conductivity bridge type 305. Infrared spectra were recorded on a
Nicolet Magna FT-IR 550 spectrophotometer in KBr pellets. H NMR and 13C NMR spectra were recorded
in DMSO-d6 using TMS as standard on a JEOL FX-90Q spectrometer. Electronic spectra in
dimethylsulphoxide were recorded on a UV-160A Shimadzu spectrophotometer. X-ray powder diffraction
spectra of the compounds were obtained on the Philips model P.W. 1840 automatic diffractometer using
Fe(K) target with Mg filter. The wavelength used was 1.9373 A and the reflections from 5 -65A were
recorded. The mass spectra of the compounds were recorded on a JEOL FX 102/ DA 6000 mass
spectrometer/data system using Argon/Xenon (6 KV, 10 mA) as the FAB gas. m-Nitrobenzyl alcohol was
used as the matrix. Manganese was estimated gravimetrically. Nitrogen was estimated by Kjeldahl's method.
Carbon and hydrogen analyses were performed at Central Drug Research Institute, Lucknow.
235
Vol. 1, Nos. 3-4, 2003 Fertility inhibitors .Macrocyclic Complexes ofBivalent Manganese
Synthetic, Spectroscopic and Medicinal Approach
RESULTS AND DISCUSSION
A series of sixteen- to eighteen-membered tetraazamacrocyclic ligands and their manganese (II)
complexes were derived by the condensation of phthalic acid with 1,2-diaminoethane or 1,3-diaminop'opane
in the presence of condensing reagents dicyclohexylcarbodiimide and 4-dimethylaminopyridine. The
reactions proceeded as given in equation 1.
2 H2N-R-NH2 + 2 HO--C6H4--OH dicyclohexylcarbodiimide
4-dimethylaminopyridine
{(HN-R-NH )} {- -C6H4- -}
LiAIH4
-4H20
H H
MnX2.4H20
-4HO
H H
[Mn{(HN-R-NH) {--C6H4--}x2]
H H
where:
R 1,2- C2 H4 or 1,3- C3 H6
X (NO3) or (CH3 COO) (Eqn. 1)
All the complexes are coloured solids. They are soluble in most of the organic solvents. The experimental
conductivity values measured for 103M solutions in anhydrous dimethylformamide are in the range 16-32
ohln-I
cm tool1
showing that they are non-electrolytes. This indicated that the anions are coordinated to the
manganese metal in these complexes. The physical properties and analytical data of the complexes are given
in Table 1.
236
R. V. Singh et al. Bioinorganic Chembstry andApplications
>., >-, 0 0
Z Z 0 0 0 0
0 0 Z Z Z Z Z Z
0 0
237
Vol. 1, Nos. 3-4, 2003 Fertility Inhibitors Macrocyclic Complexes ofBivalent Manganese
Synthetic, Spectroscopic and Medicinal Approach
Infrared Spectra:
A comparative study of the IR spectra of the tetraamides, tetraazamacrocyclic ligands and their
corresponding manganese (II) complexes confirmed the formation of the macrocyclic complexes with the
proposed coordination pattern. The ligands DTTD and DTTD show the absence of bands corresponding to
primary amino and hydroxy groups, which confirms their involvement in the formation of tetraamide
macrocycles. The presence of four bands in the regions 1640-1648, 1485-1489, 1230-1239 and 620-632 cm1
is due to amide I, amide II, amide III, and amide IV bands respectively/16/. This provides strong evidence
for the presence of a closed cyclic product. A single sharp band observed for amide ligands DTTD and
DTTD in the region 3275-3289 cm1
is due to v (NH) of amide group. Strong and sharp absorption bands
that appear in the regions 2800-2925 and 1400-1450 cm
-in all the complexes may be ascribed to the C-H
stretching and bending vibrations, respectively/17/. In the IR spectra of tetraazamacrocyclic ligands TTD
and TTD2, it is observed that the amide bands have been found absent on comparing with DTTD and
DTTD2.
In the spectra of macrocylic complexes of manganese (II) as compared to their analogous metal free
tetraaza ligands a slight negative shift in the v (N-H) band that appeared in the region 3210-3235 crn
-was
observed. It is ascribed to the coordinated N-H stretching vibration. The shift to the lower frequency ofv (N-
H) mode, along with the appearance of a new band in the region 303-329 cm1
assignable to the v(Mn-N)
vibrations, suggested that the amide nitrogen is coordinated to the manganese/18,19/. The infrared spectral
data of the ligands and their manganese(If) complexes are reported in Table 2. The nitro complexes exhibit a band
in 243-258 cm region assignable to v (Mn-O) ofONO2 group/20,21/.
H and 13C NMR Spectra
The bonding pattern in the ligands has also been sustained by the proton magnetic resonance spectra. The
ligands DTTD and DTTD do not show any signal corresponding to primary amino and alcoholic protons
suggesting that the proposed macrocyclic skeleton has been formed through condensation reaction. Broad
signals exhibited by the ligands DTTD and DTTD at 6 8.44 and 8.47 ppm, respectively, are due to the
amide proton (CO-NH). The signals due to the methylene protons 5 3.39- 3.68 ppm were also assigned for
the ligands. A multiplet in the region 6 1.98 2.04 ppm may be attributed to the central methylene protons,
-C-CH-C-. A multiplet ofaromatic protons was observed at 5 7.28 8.05 ppm in the spectra of all the ligands.
The conclusions drawn from the IR and H NMR spectra are parallel with the carbon-13 spectral data
regarding the authenticity of the proposed skeleton. H NMR and 13C NMR spectral data of the ligands are
listed in Table 3.
Electronic Spectra
The electronic spectra of the complexes display weak absorption bands in the regions 17245-16930,
23827-23204 and 27020-26035 cm for 6Aig 4ylg 6Alg 4T2g and 6Alg 4Tlg respectively. These data
are in fair agreement with the octahedral geometry for the Mn(II) complexes/22/.
238
R. V. Singh et al. Bioinorganic Chemistry andApplications
0 0
239
Vol. 1, Nos. 3-4, 2003 brtiliO, Inhibitors Macrocyclic Complexes ofBivalent Manganese
Synthetic, Spectroscopic andMedicinal Approach
240
R. l,: Singh et al. Bio&organic ChemistIy andApplications
Magnetic Moment"
The lab values for the complexes observed at 5.82-5.89 BM are within the range required for an
octahedral geometry/23/.
Mass Spectrum
The mass spectrum of the compound [Mn(TTDZ)(CH3COO)2] shows the molecular ion peak at m/z =526.
The prominent fragments are at 454 for [Mn(C23 H30N204)]+,422 for [Mn(C18H30N404)]+
and 382 for
[Mn(C20H2204)]+
due to the loss of C3HsN2, CH and C6HI6N4, respectively. Another peak appeared at m/z
408 due to the loss oftwo acetate groups from the parent ion.
X-ray Diffraction Studies
The X-ray diffraction analysis of the compound [Mn(TTD)(NO3)2] confirms the orthorhombic crystal
system for this derivative having unit cell dimensions, a=13,.0202, b 22.4347 and c=17.0650 and
et=[=7=90.Miller indices h, k and are given in Table 4. The structure shown in Figure may be assigned
to the complexes based on the preceeding spectral studies.
Where. X NO or CHCOO
n=2or3
H--i' X I-H
H NH
"'1/(CH2)n
(H2
,nN.../"
M n
H NH
H----C.
/C'---H
Fig.
241
Vol. 1, Nos. 3-4, 2003 Fertilio Inhibitors Macrocyclic Complexes ofBivalent Manganese
S),nthetic, ,Spectroscopic and Medicinal Approach
Table 4
X-Ray Powder Diffraction Data ofthe Compound/Mn(TTD2)(NO,02/
Peak No.
1.
2.
3.
4.
5.
6.
7.
8.
9.
10.
ll.
12.
13.
14.
15.
16.
17.
18.
19.
20 (deg.) d spacing h k
(obs.) (obs.)
12.4 8.9593 3
18.6 5.9942 0 3 2
21.0 5.9942 0 5 5
22.0 5.3155 2 3
23.2 5.0767 4
24.7 4.8174 2 4
25.4 4.5290 5 3
26.2 4.2739 0 2 5
27.2 4.1195 0 5 4
28.0 4.0041 2 5
29.2 3.8429 4 4
31.6 3.5577 0 6 4
32.8 3.4309 5 5
33.0 3.4107 5 3 0
33.74 3.3380 3 8 0
34.8 3.8393 2 7
36.86 3.0688 5 5 2
37.4 3.0213 3 9
38.6 2.9308 0 9 4
Refined values a=13.0202 ct=[3==90
b 22.4347
c 17.0650
(Orthorhombic system)
242
R. V. Sinh et al. Biohorganic Chemistty andApplications
Biological Screening"
To assess the biological potential of the ligands and their macrocyclic complexes, laboratory and field
experiments have been conducted. The following techniques have been used for the antimicrobial activities
ofthese compounds.
Antifungal Activities
1. Poisoned Food Technique: The antifungal activity was evaluated by the food poisoned technique/24/.
2. Spore Germination Method/25/:
The spore germination test includes depositions of chemicals on a slide, evaporation to dryness and
addition of a drop ofwater containing spore ofthe test fungus.
A drop of spore suspension was placed on a clear slide. The number of spores in the drop under low
power magnification was determined and the suspension was adjusted to obtain nearly ten spores per
microscopic field. These slides were incubated for 12 hours at 25C in petri dishes working as moisture
chambers. Slides were put in such a position that spore drops keep hanging. After incubations of 12 hours,
spore germinations were counted. Each treatment was replicated thrice. In this technique total number of
spores, number of germinated spores and number of ungerminated spores were counted. LDs0 values were
calculated to compare the activity of the ligands along with their chelates by plotting graphs between the
concentration of ligand vs complex with the number of germinated spores. The organisms selected fir this
method are; Alternaria alternata and Helminthosporium gramineum.
Antibacterial Activity
Inhibition Zone Technique/26/:
In this technique sterilized hot nutrient agar medium and 5 mm diameter paper discs of Whatman No.
were used. The agar medium was poured into the petri plates. After solidifications, the petri plates were
stored in inverted position so that there was condensation of water in the upper lid. Solutions of test
compounds in methanol in 500 and 1000 ppm concentrations were prepared in which discs were dipped in
solution of the test sample placed on seeded plates. The petri plates having these discs on the seeded agar
should first be placed at low temperature for two or four hours to allow for the diffusion of chemicals before
being incubated at suitable optimum temperature 28 + 2C for 24-30 hours. After the expiry of their
incubation period, the zone of inhibition associated with the treated disc was measured in mm. The
compounds were tested against Pseudomonas cepacicola (-), Xanthomonas compestris (-), Escherichia coli
('-) and Staphylococcus aureus (+).
Antlertility Activity
The antifertility activity of the ligands and their complexes was studied on male albino rats.
ttealthy, adult male albino rats of sprague dawley strain were used in the present investigations. The rats
were divided into seven groups of eight animals each. The group A served as vehicle (olive oil) treated
control. In the group B, iigand D'FTD 25 mg kg body weight suspended in 0.2 mL olive oil was given
243
Vol. 1, Nos. 3-4, 2003 Fertility Inhibitors Macrocyclic Complexes ofBivalent Manganese
A),nthetic, Spectroscopic and Medicinal Approach
orally for a period of forty five days. The animals of groups D and G received the same dose of compounds,
respectively, for a similar period. The animals were screened for fertility test and autopsied for detailed
pathological and biochemical studied. Reproductive organs were excised, blotted free of blood, weighed and
fixed in Bouin's fluid for histological studies.
The sperm motility and density of Cauda epididymal spermatozoa was assessed. The protein, sialic acid
/27/and fructose were determined/27/. The activity of acid phosphate was estimated. The vaginal smear or
vaginal plug confirmed the positive mating. The females were checked for implantation after 16 days of
pregnancy. The results were analysed with the help of student 't' test.
RESULTS AND DISCUSSION
Antifungal and Antibacterial Screening
Antifungal and antibacterial activities of representative ligands and their manganese complexes have been
screened in Tables 5, 6 and 7. The results howed that there is considerable increase in the toxicity of the
complexes as compared to the ligands.
On giving a closer look at these results a common feature which appears is that the bioactivity enhances
undergoing chelation. This can be well ascribed to Tweedy's chelation theory/28/. According to this, the
chelation reduces the polarity ofthe central atom mainly because of partial sharing of its positive charge with
the donor groups and possible -electron delocalisation over the whole chelation ring. This increases the
lipophilic nature of the central manganese atom, which subsequently favours its permeation through the lipid
layer ofthe cell membrane.
Solubility and concentration of the compounds play a vital role in ascertaining the extent of inhibition.
The biological potency of these compounds may be attributed to their ability to inactivate various cellular
enzymes which play a vital role in different metabolic pathways of these microorganisms. It has been
proposed that the ultimate action of these compounds is the denaturation of one or more proteins of the cell
as a result of which normal cellular processes are impaired. It may also be postulated that these complexes
might act as uncoupling agents of oxidative phophorylation of ADP to ATP /29/. These agents are less
effective for bacteria.
Representative ligands and their manganese complexes have been screened in vivo to study their potency
on crop plants guar (guar blight by Alternaria cymopsidal) mung (Cercospora leafspot) using l:'ercent
Disease Incidence (PDI) technique.
The Percent Disease Incidence (PDI) is the area covered on foliage by specific disease and was calculated
by using the following formula:
Percent Disease Incidence
Numberof infected plants
Total number of plants observed
xl00
Percent Disease Control
Numberof treated plants PDI in untreated plant
PDI in untreated plants
100
244
R. V. Singh et al. Bioinorganic ChemisoT andApplications
245
Vol. 1, Nos. 3-4, 2003 Fertilio, Inhibitors Macrocyclic Complexes ofBivalent Manganese
Synthetic, Spectroscopic and Medicinal Approach
Table 6
Antifungal Screening Data ofthe Ligands and their Macrocyclic Complexes ofManganese(II).
Compound COllC.
ppm Total no.
of spores
50 10
100 10
DTTD
2O0 10
400 10
50 10
100 10
DTTD
200 10
400 10
50 I0
100 10
TTD
200 10
400 10
50 10
100 10
TTD
2O0 10
400 10
50 10
100 10
[Mn(TTD)(NO3)]
200 10
400 10
50 10
100 10
[Mn(TTD2)(NO3)2] 20O 10
400 I0
Alternaria alternata Helm#ffhosporium gram#eum
No. of No. of Total no. No. of No. of
germinated ungerminated of spores germinated ungerminated
spores spores spores spores
9 12 8 4
7 3 12 4 8
5 5 12 3 9
2 8 12 2 10
7 3 12 7 5
5 5 12 5 7
2 8 12 4 8
9 12 2 10
7 3 12 7 5
6 4 12 5 7
3 7 12 4 8
9 12 2 10
6 4 12 2 10
5 5 12 5 7
3 7 12 7 5
2 8 12 9 3
8 2 12 7 5
6 4 12 4 8
4 6 12 3 9
3 7 12 11
6 4 12 7 5
4 6 12 4 8
3 7 12 3 9
2 8 12 11
Comparison of LDso Values (ppm) of Ligands and their Macrocyclic Complexes of Mangnese(ll).
Compound Alternaria alternata Helm#thosporium gramineum
DTTD 200 75
DTTD 100 70
TTD 130 67
TTD 100 125
Mn(TTD)(NO3)2] 120 60
[Mn(TTD2)(N03)2] 70 65
246
R. V. Singh et al. Bio&organic Chemistty andApplications
247
Vol. 1, Nos. 3-4, 2003 FertiliO Inhibitors Macrocyclic Complexes ofBivalent Manganese
Synthetic, Spectroscopic and Medicinal Approach
Field experiments were laid out in randomized block design with three replications. The crops were raised
in each plot. Compounds with a standard fungicides, bavistin [2-methoxycarbamyl) benzimidazole] were
tried in addition to check (water spray).
After sowing of 25 days, the plants were inoculated artificially by spraying the conidial suspension. The
conidial suspension was prepared by crushing the infected leaves in water. The inoculation was done late in
the evening.
The first spray of the respective fungicide was given when lesions were first seen and spraying was
repeated after 10 days. Disease intensity was recorded 10 days after the second spray. The data was analysed
statistically and Percent Disease Control was worked out. It was observed that, although all the fungicides
were significantly at par with each other and different from check, yet the performance of the complexes are
higher than their respective ligands.
Antifertility Activity:
The testicular morphology, testicular sperm density, sperm motility, density of cauda epid!dymal
spermatozoa and fertility in mating trials and biochemical parameters of reproductive organ.,; with
tetraamides and their manganese complexes in vivo were examined and discussed.
The results show that the ligands alone were able to inhibit fertility but due to the added synergistic
effects of the manganese complexes, their activity becomes enhanced. The results are grouped under the
following headings:
Bo@ and Organ Weights
Body weights of rat were not affected after their manganese complexes were administrated. However, the
weights oftestes, epididymis, seminal vesicle and ventral prostate were significantly decreased [Table 8].
Fertility Test
The sluggish motile spermatozoa was unable to fertilize normal cyclic females. The test was 68 to 92%
negative in rats treated with the compounds.
Sperm Motility
The sperm motility declined significantly after treatment with compounds.
Sperm Density
The sperm density in testes and cauda epididymis declined significantly after treatment [Table 9].
Biochemical Changes
Total protein and sialic acid contents of testes, epididymis, ventral prostate and seminal vesicle were
depleted significantly after treatment with ligands and their complexes. The acid phosphate levels of testes,
epididymis and ventral prostate were also reduced significantly. A significant decrease in seminal vascular
248
R. V. Singh et al. Bioinorganic Chemistly andApplications
0
o Vl Vl Vl
249
Vol. 1, Nos. 3-4, 2003 Fertility hhibitors Macrocyclic Complexes ofBivalent Manganese
Synthetic; Spectroscopic and Medicinal Approach
"Jl-
V! VI
250
R. V. Singh et al. Bioino1Lganic ChemistIy andApplications
fructose contents was also noticed whereas the testicular cholesterol contents were increased significantly
after the treatment with various compounds [Tables 10 and 11].
Discussion
The present study revealed that administration of ligands and their manganese complexes caused a
significant reduction in the weights of testes and other sex accessory glands. The prostate and seminal vesicle
are well documented androgen dependent processes /31/. Sperm motility is considered as an important
parameter in evaluating the fertility potential/32/. The tetraamide ligands and their manganese complexes
significantly reduce the fertility of male rats. Since a number of androgen sensitive parameters (protein, sialic
acid, fructose, acid phosphatase and total cholesterol) in target organs were found to be altered by these
complexes, it is probable that the structure and function of epididymis and other sex accessory organs are
changed.
Manganese chloride causes testicular germ cells in rats and rabbits /33/ and decreased libido and
impotency in a man occupationally exposed to manganese/34/. Our findings indicate that the ligand TTD
and its manganese complex have a more pronounced effect of fertility on various biochemical parameters of
reproductive organs as compared to other ligands and complexes discussed in this paper.
ACKNOWLEDGEMENT
One of the authors (AC) is thankful to CSlR New Delhi for financial assistance in the form of SRF vide
Grant No. 9/149(288)/2K2, EMR1.
REFERENCES
1. H. Sigel, Metal Ion in Biological Systems, Vol. 2; Mixed-Ligand Complexes, Marcel Dekker, Inc., New
York, 1993.
2. T.A. Khan, Syed S. Hasan, A.K. Mohamed, K.S. Islam and M. Shakir, Synth. React. Inorg. Met-Org.
Chem., 30(5), 815 (2000).
3. A.C. Hiremath, K.M. Reddy and M.B. Halli, Asian, J. Chem., 5, 35 (1993).
4. F.A. Catton and G.Willkinson, Advanced Inorganic Chemistry, John Wiley and Sons, New York, 1988;
p. 730.
5. M.W. Hosseini, J.M. Lehn, S.R. Duff. K. Gu and M.P. Mertes, J. Org. Chem., 52, 1662 (1987).
6. R.M. lyan, J.S. Brandshaw, S.A. Neilsen, J.D. Lamb, J.J. Christensen and D. Sen, Chem. Rev., 85, 271
(1985).
7. X.M. Chem, Z. T. Xu and X.C. Huang, J. Chem. Soc., Dalton Trans, 1994, 2331.
251
oI. 1, Nos. 3-4, 2003 Fertility bhibitors Macrocyclic Complexes ofBivalent Manganese
S),thetic, Spectroscopic and Medicinal Approach
VI VI Vl
252
R. V. Singh et aL Bioinorganic Chemistry andApplications
253
Vol. 1, Nos. 3-4, 2003 Fertility hThibitors Macrocyclic Complexes ofBivalent Manganese
Synthetic, Spectroscopic and Medicinal Approach
8. S. Ozawa, Y. Watanabe, S. Nakashima, T. Kistogawa and I. Morishima, J. Am. Chem. Soc., 116, 634
(1994).
9. P.I. Pessiki, S.V. Khangulov, D.M. Ho and G.C. Dismukes, J. Am. Chem. Soc., 16, 891 (1994).
10. K. Weigharadt, Angew Chem. Int. Ed. Engl., 28, 1153 (1990).
11. A. Willing, H. Folman and G. Aulig, Eur, II, Biochem. Soc., 170, 603 (1988).
12. A.S. Goldstein, R.H., R.H. Beer, R.S. Drago, J. Am. Chem. Soc., 116 2424 (1994).
13. J.W. Gobdes and W.H. Armstrong, Inorg. Chem., 31,368 (1992).
14. M.L. Ludwig, K.A. Pattridge and W.C. Stallings, Metabolism and Enzyme Function, Academic Press,
New York, 405, 1986.
15. R.D. Guieles, V.K. Yachanadra, A.E. Mc. Dermott, J.L. Cole, S.L. Dexheimer, R.D., Briti, K. Sauer
and M.P. Klein, BiochemisOy, 29, 486 (1990).
16. A. Chaudhary, A. Phor and R.V. Singh, IndianJ. Chem., 41A, 2536 (2002).
17. A. Chaudhary, S. Dave, R. Swaroop and R.V. Singh, Indian J. Chem., 40A, 757 (2001).
18. M.B.H. Howladar, M.S. Islam and M.R. Karim, Indian J. Chem., 39A, 407 (2000).
19. D.M. Adam and J.B. Carrool, J. Chem. Soc., A, 1968, 1299.
20. R.H. Balundi and A. Chakravorty, Inorg. Chem., 12, 1981 (1975).
21. A.R. Katrityky, A.R. Hands and R.A. Jones, J. Chem. Soc., 3165 (1958).
22. P.S. Mane, S.G. Shrokdar, B.R. Arbad and T.K. Chondheker, Indian J. Chem., 40A, 648 (2001).
23. R.A. Lal, A. Kumar and J. Chakravorty, Indian J. Chem., 40A, 422 (2001).
24. A. Chaudhary, S. Dave, R.K. Saini and R.V. Singh, Main Group Met. Chem., 24, 217 (2001).
25. S. Belwal, S. C. Joshi and R.V. Singh, Main Group Metal Chem., 20, 313 (1997).
26. A. Chaudhary and R.V. Singh, Metal Based Drugs, 8, 315 (2002).
27. K. Sharma, S.C. Joshi and R.V. Singh, Metal Based Drugs, 7, 237 (2000).
28. A. Bansal, R.D. Singh and R.V. Singh, Metal Based Drugs, 7, 211 (2000).
29. S. Belwal, R.K. Saini and R.V. Singh, Indian J. Chem., 37A, 245 (1998).
30. S.C. Vyas Fungicides, Vol. 1, 2, 3, Oxford Indian Book House, New Delhi, 1995.
31. T. Mann and C. Mann Lutwak, in: Male Reproduction Function and Semen, Springer Verlag, Berlin,
New York, 1981; p. 139.
32. G. Gupta, A.K. Srivastava and B.S. Shetty, Indian J. Exptl. Biol., 33, 281 (1995).
33. R.H. Dixon, Reproductive Toxicity, Raven Press, New York, 309 (1985).
34. P. Schulfer, H. Oyanguren, V. Maturana, A. Valenzuela, E. Cruz, V. Plaza, E. Schmid and R. Haddad,
Industrial Medicine and Surgery, 26, 167 (1957).
254

				

